# Supplementary material for: Health System Barriers and Facilitators to Delivering Additional Vaccines through the National Immunisation Programme in China: A Qualitative Study of Provider and Service-User Perspectives
Source: Vaccines (Basel). 2021 May 8;9(5):476. doi: 10.3390/vaccines9050476 (PMC8151436; doi:10.3390/vaccines9050476)
Supplement: Supplementary file 1 [file vaccines-09-00476-s001.zip › vaccines-1148295-supplementary.pdf]

Table S1. EPI-provided and non-EPI vaccines available in China.

EPI Vaccines	Non-EPI Vaccines
AVA (Anthrax vaccine adsorbed)	EV-71 (Enterovirus 71) vaccine
HFRS (Haemorrhagic fever with renal syndrome) vaccine	Hib (Haemophilus influenzae type b) vaccine
BCG (Bacillus Calmette-Guerin) vaccine	HepE (Hepatitis E) vaccine
DTP (Diphtheria, tetanus, and pertussis) vaccine	HPV (Human papillomavirus) vaccine
DT (Diphtheria and tetanus) vaccine	Influenza vaccine
HepA (Hepatitis A) vaccine	Leprosy vaccine
HepB (Hepatitis B) vaccine	OCV (Oral cholera vaccine)
JE (Live attenuated Japanese encephalitis) vaccine	PCV (Pneumococcal conjugate vaccine)
Leptospirosis vaccine	DTaP-IPV-Hib (Diphtheria, tetanus, pertussis, polio, haemophilus influenzae Type b) vaccine
MMR (Measles, mumps and rubella) vaccine	Rabies vaccine
MPV-A (Group A meningococcal polysaccharide vaccine）	RV (Rotavirus vaccine)
MPV-AC (Group A and group C meningococcal polysaccharide vaccine)	Tetanus vaccine
MR (Measles-rubella) vaccine	Varicella vaccine
OPV (Oral polio vaccine)	

Table S2. Main socio-demographic indicators of study provinces in 2019.

Indicator	Guangdong (Eastern)	Henan (Central)	Sichuan (Western)
Per capita GDP (RMB)	94,172	56,388	55,774
Life expectancy (year)	76.5	74.6	74.8
Maternal mortality rate (1/100,000)	7.3	9.2	10.9
Population (10,000 persons)	11,521	9,640	8,375
Per capita disposable income of urban households(RMB)	48,117.6	34,201.0	36,153.7
Per capita disposable income of rural households(RMB)	18,818.4	15,163.7	14,670.1

Note: Data source China Health Statistics Yearbook (2020).
